# The X-Linked Tumor Suppressor TSPX Regulates Genes Involved in the EGFR Signaling Pathway and Cell Viability to Suppress Lung Adenocarcinoma

**DOI:** 10.3390/genes16010075

**Published:** 2025-01-11

**Authors:** Tatsuo Kido, Hui Kong, Yun-Fai Chris Lau

**Affiliations:** Division of Cell and Developmental Genetics, Department of Medicine, Veterans Affairs Medical Center, and the Institute for Human Genetics, University of California, San Francisco, CA 94121, USA; tatsuo.kido@ucsf.edu (T.K.); konghui@njmu.edu.cn (H.K.)

**Keywords:** TSPX, DENTT, lung adenocarcinoma, non-small cell lung cancer, RNA-seq, X-linked tumor suppressor, EGFR signaling pathway

## Abstract

**Background:** TSPX is an X-linked tumor suppressor that was initially identified in non-small cell lung cancer (NSCLC) cell lines. However, its expression patterns and downstream mechanisms in NSCLC remain unclear. This study aims to investigate the functions of TSPX in NSCLC by identifying its potential downstream targets and their correlation with clinical outcomes. **Methods**: RNA-seq transcriptome and pathway enrichment analyses were conducted on the TSPX-overexpressing NSCLC cell lines, A549 and SK-MES-1, originating from lung adenocarcinoma and squamous cell carcinoma subtypes, respectively. In addition, comparative analyses were performed using the data from clinical NSCLC specimens (515 lung adenocarcinomas and 502 lung squamous cell carcinomas) in the Cancer Genome Atlas (TCGA) database. **Results**: TCGA data analysis revealed significant downregulation of TSPX in NSCLC tumors compared to adjacent non-cancerous tissues (Wilcoxon matched pairs signed rank test *p* < 0.0001). Notably, the TSPX expression levels were inversely correlated with the cancer stage, and higher TSPX levels were associated with better clinical outcomes and improved survival in lung adenocarcinoma, a subtype of NSCLC (median survival extended by 510 days; log-rank test, *p* = 0.0025). RNA-seq analysis of the TSPX-overexpressing NSCLC cell lines revealed that TSPX regulates various genes involved in the cancer-related signaling pathways and cell viability, consistent with the suppression of cell proliferation in cell culture assays. Notably, various potential downstream targets of TSPX that correlated with patient survival (log-rank test, *p* = 0.016 to 4.3 × 10^−10^) were identified, including EGFR pathway-related genes *AREG*, *EREG*, *FOSL1*, and *MYC*, which were downregulated. **Conclusions**: Our results suggest that TSPX plays a critical role in suppressing NSCLC progression by downregulating pro-oncogenic genes, particularly those in the EGFR signaling pathway, and upregulating the tumor suppressors, especially in lung adenocarcinoma. These findings suggest that TSPX is a potential biomarker and therapeutic target for NSCLC management.

## 1. Introduction

Lung cancer is the leading cause of cancer death worldwide, approximately 1.76 million death each year (18.4% of the total cancer death) [1]. Most lung cancer cases (~85%) are classified as non-small cell lung cancer (NSCLC) consisting of three major subtypes, i.e., adenocarcinoma, squamous cell carcinoma, and large cell carcinoma [2,3]. NSCLC is considered a biologically aggressive cancer type with rapid growth and progression and a low 5-year survival rate of approximately 20% overall [4]. The key issues in NSCLC are its tumor heterogeneity and highly variable responses to clinical treatments [2,5,6]. Thus, effective identifications of different histological subtypes and prognostic markers such as driver mutations would greatly improve the outcomes and survival of patients [3,6,7]. For instance, the activating mutations of the epidermal growth factor receptor (EGFR) gene (EGFR-mut) have been observed in 15% (Europe) to 62% (Asia) of lung adenocarcinoma cases [2,7]. Excessive activation of the EGFR signaling cascade is key for the development of NSCLC [2,6]. Patients harboring EGFR-mut are responsive to the treatment of EGFR tyrosine kinase inhibitors (EGFR-TKIs), hence EGFR-TKIs are recommended as a first-line treatment plan for EGFR-mut patients [2,3,7]. Identifying prognostic biomarkers could help not only in further classification but also provide an opportunity to elucidate their biological roles in NSCLC, thereby improving personalized precise therapies for patients [3,6,8,9].

*TSPX* (also known as *DENTT*, *CDA1*, and *TSPYL2*) is an X-linked tumor suppressor gene originally identified as a TGF-β responsive member of the TSPY/TSPY-like/SET/NAP-1 superfamily in a human NSCLC cell line [10]. While it is ubiquitously expressed and likely serves as a housekeeping gene [11,12], it is frequently downregulated in various types of cancer, including liver cancer, glioma, prostate cancer, thyroid cancer, and lung cancer [13,14,15,16,17]. Downregulation of TSPX has been linked to aberrant DNA hypermethylation, as treatment with the demethylating agent 5-aza-2′deoxycytidine restores its expression in NSCLC and glioma cell lines [15,18]. In addition, TSPX mutations in endometrial tumors and uterine leiomyomas have been reported [19,20]. TSPX protein harbors a SET/NAP-domain critical for chromatin/histone modification, gene regulation, and cell-cycle regulation [21,22,23,24,25]. Previous studies, including ours, have demonstrated that TSPX inhibits cyclin-B/CDK1 kinase activity, activates the p53 pathway, suppresses oncogenes such as *MYC* and *MYB*, inhibits sirtuin 1 (SIRT1), and represses androgen receptor transactivation [16,17,26,27,28,29,30]. It is also an essential component of the REST/HDAC repressor complex, suggesting a multi-functional role in gene regulation [26]. However, the downstream targets of TSPX in lung cancer are still largely unknown. Furthermore, the correlation between TSPX expression and clinical features of lung cancer, such as cancer grades, prognosis, and survival remain to be elucidated.

In the present study, we examined the expression levels of TSPX in the transcriptomes of lung adenocarcinoma (*n* = 515) and lung squamous cell carcinoma (*n* = 502) specimens in the Cancer Genome Atlas (TCGA) [31]. Our results demonstrated that TSPX expression is downregulated in cancer specimens compared to adjacent non-tumor lung specimens, as determined by Wilcoxon matched pairs signed rank test. Notably, higher TSPX expression levels were significantly correlated with lower tumor grades and better survival rates in lung adenocarcinoma patients, as assessed by log-rank test. To evaluate its potential effects in cellular properties, TSPX was overexpressed in the lung adenocarcinoma cell line A549 and the lung squamous cell carcinoma cell line SK-MES-1, followed by analysis of cell proliferation and cell survival. The results showed that TSPX overexpression inhibited cell proliferation in both A549 and SK-MES-1 cell lines. Consistently, transcriptome and pathway enrichment analyses of the TSPX-overexpressing A549 and SK-MES-1 cells, performed using RNA-seq and DAVID bioinformatics resources [32], indicated that TSPX regulates genes involved in oncogenic and other key signaling pathways, including NF-kB, Wnt, and MAPK signaling pathways. Further comparative analyses of the transcriptomes of A549 and SK-MES-1 cells overexpressing TSPX, and clinical lung adenocarcinoma specimens with high or low TSPX expression from TCGA datasets identified potential downstream target genes of TSPX associated with patient survival. These targets included downregulated genes such as the EGFR ligands AREG and EREG, the oncogenic transcription factors MYC and FOSL1, the apoptosis inhibitor BIRC3, and the receptor ligands DKK and PLAU. Of note, AREG, EREG, FOSL1, MYC, and PLAU are involved in the EGFR signaling pathway, which is a key pathway in lung cancer development [2,6]. Additionally, the tumor suppressor CACNA2D2 was upregulated. Since high TSPX expression levels are associated with lower tumor grades and better survival in lung adenocarcinoma patients, our findings suggest that TSPX plays a critical role as an X-located tumor suppressor that represses the development and progression of lung adenocarcinoma. Its reduced expression could serve as an indicator/prognostic marker of the aggressiveness of lung adenocarcinoma. This is the first study to establish a correlation between TSPX expression levels and clinical outcomes, and also provide a comprehensive analysis of TSPX downstream genes and pathways in NSCLC. These findings have the potential to improve personalized and precise therapeutic strategies for NSCLC patients.

## 2. Materials and Methods

### 2.1. Cell Culture and Lentiviral Transduction

The human lung adenocarcinoma A549 cell line and lung squamous cell carcinoma SK-MES-1 cell line were obtained from ATCC (Manassas, VA, USA) through the Cell and Genome Engineering Core (CGEC) at the University of California San Francisco (UCSF) and verified by short tandem repeat (STR) analysis [33]. The cells were thawed and cultured in RPMI 1640 medium or Dulbecco’s modified Eagle’s medium (DMEM) containing 10% Tet-system approved fetal bovine serum (FBS, Clontech/Takara Bio USA, Mountain View, CA, USA) and an antibiotics cocktail (100 U/mL penicillin and 100 µg/mL streptomycin). They were used immediately in experiments described in this study. *FUW-tetO-TSPX*, a lentiviral vector capable of expressing both full-length TSPX and EGFP under the control of doxycycline (Dox), was prepared as described previously [16]. *FUW-tetO-EGFP*, a lentiviral vector for expression of EGFP alone, was used as a negative control. The generation of replication-incompetent lentiviruses followed the methods previously reported [16,29]. Cells were transduced with lentiviral particles containing the expression vectors, *FUW-tetO-TSPX* or *FUW-tetO-EGFP* with *FUW-M2rtTA*. The transduced cells were cultured in the absence of Dox until analysis. To induce the expression of TSPX and/or EGFP in the transduced cells, cells were cultured in the presence of 0.5 µg/mL Dox (Sigma-Aldrich, St. Louis, MO, USA). The Institutional Bio Safety Subcommittee has reviewed and approved all recombinant DNA and lentiviral transduction experiments.

The migratory properties and morphological changes were measured by a scratch wound assay [34]. Briefly, cells were plated at 5 × 10^5^ cells/60 mm dish and cultured for 24 h in the absence of Dox. Before treatment with Dox, dishes were scratched at 3 sites with a sterile 1000 μL pipette tip. Cells were cultured with fresh medium with or without 0.5 µg/mL Dox. Images of the scratched areas were recorded at 0 and 48 h after scratching and changes at a representative site are shown in the figure.

### 2.2. Cell Proliferation Assay

For cell proliferation analysis, cells were seeded at 1000 cells/well in 96-well plates and cultured in the presence or absence of 0.5 µg/mL Dox. The cell viability was analyzed at the indicated time points using the CellTiter 96 Aqueous One Cell Proliferation Assay kit (Promega, Madison, WI, USA), according to the manufacturer’s instructions. Each experimental group consisted of 6 wells per time point, and statistical comparisons were performed using Student’s *t*-test for each time point. Differences with a *p*-value < 0.05 are considered statistically significant.

### 2.3. Immunofluorescence and Annexin-V Binding Assay

Immunofluorescence was performed after fixation with 4% paraformaldehyde and permeabilization with methanol as described previously [16], using anti-GFP goat IgG (Abcam, Cambridge, MA, USA) and anti-TSPX mouse monoclonal IgG (generated in the lab). Annexin-V binding assay was used to detect apoptotic and dead cells, as previously described [16]. The experiments were repeated at least twice to verify the results.

### 2.4. Western Blot

Western blot was performed as described previously [35], using anti-GFP goat IgG, anti-TSPX rabbit IgG (Bethyl Laboratories, Montgomery, TX, USA), and anti-βactin mouse monoclonal IgG (clone AC-15, Sigma-Aldrich). In brief, transduced cells were cultured in 12-well plates in the presence of 0.5 µg/mL Dox for 24 h, lysed in 100 µL SDS sample buffer, and denatured at 100 °C for 10 min. Ten µL of each sample were subjected to Western blot analysis. The experiment was performed in triplicate, and a representative result is shown in the figure.

### 2.5. RNA Preparation and RNA-Seq Transcriptome Analysis

Total RNA was isolated from the A549 cells and SK-MES-1 cells and subjected to RNA-seq transcriptome analysis using the Illumina NextSeq 500 sequencer, as previously described [16,29]. In brief, each experimental group consisted of 3 wells in a 6-well plate, with cells cultured in the presence of 0.5 µg/mL Dox for 24 h. Sequencing libraries were independently prepared from cells in each well and sequenced separately, representing biological triplicates. After initial quality assessment by FastQC program (version 0.11.4) [36], the sequence reads were mapped onto the human reference genome GRCh37/hg19 using the TopHat program (version 2.1.0) [37]. The mapped reads were summarized and calculated to the count reads that could be associated with the expression levels using the featureCounts program (version 1.6.0) [38]. Normalization of data and differential gene expression analysis were performed using a TCC/edgeR software package (version 1.46.0) [39]. Genes representing changes with TCC/edgeR software analysis FDR < 0.005, Student’s *t*-test *p*-value < 0.05, Log_2_(gene expression level) > 4, and |Log_2_(fold change)| > 0.85 were considered as differentially expressed genes (DEGs). Pathway enrichment analyses were performed using DAVID bioinformatics resources [32]. The pathways with a *p*-value < 0.05 are considered statistically significant. The datasets used and/or analyzed for the present study are available on request.

### 2.6. Dataset and Data Mining Analysis of Lung Adenocarcinoma and Lung Squamous Tumor Specimens from the TCGA Database

The RNA-seq gene expression data and associated clinical information of lung adenocarcinoma and lung squamous cell carcinoma cases at the Cancer Genome Atlas (TCGA) data portal were downloaded from the UCSC Xena Browser [31,40]. The dataset of lung adenocarcinoma (LUAD) included 59 non-tumor samples and 515 tumor samples, and the dataset of lung squamous cell carcinoma (LUSC) included 51 non-tumor samples and 502 tumor samples. The expression levels were calculated as an RSEM normalized read count [41]. The survival information of the respective patients in the TCGA database was obtained from the Human Protein Atlas (HPA) data portal [31,42], except for the classification of the high TSPX-expressing patients and the low TSPX-expressing patients. Fifteen cases in the LUAD dataset and seven cases in the LUSC dataset lacked survival information. Statistical analyses were performed with the Prism10 program (version 10.4.1) (GraphPad Software, La Jolla, CA, USA). Differences in gene expression levels (RSEM normalized read counts) were evaluated by one-way ANOVA followed by Tukey’s multiple comparison test. Differences with a *p*-value < 0.05 were considered statistically significant.

The clinical transcriptome and patient survival data were downloaded from the public domain repository TCGA portal in a de-identified manner. The human studies were approved with a waiver by the Institutional Human Research Committee.

### 2.7. Quantitative RT-PCR (RT-qPCR) Analysis

Total RNA was isolated from the Dox-induced A549 cells and SK-MES-1 cells using TRIZOL-plus kit (Thermo Fisher Scientific/Invitrogen, Carlsbad, CA, USA), and analyzed by RT-qPCR, using GoTaq qPCR Master Mix (Promega, Madison, WI, USA) or TaqMan Fast Advanced Master Mix (Thermo Fisher Scientific), and QuantStudio3 real-time PCR detection system (Thermo Fisher Scientific). In brief, each experimental group consisted of 3 wells, with cells cultured for 24 h in the presence of 0.5 µg/mL Dox. Reverse transcription products were independently prepared from cells in each well (biological triplicate) and analyzed by quantitative PCR in technical triplicates. The expression levels of the respective genes were normalized to that of the GAPDH gene. Statistical significance was evaluated using Student’s *t*-test. Difference with a *p*-value < 0.05 were considered statistically significant. The primer sequences are described in Supplementary Table S1.

## 3. Results

### 3.1. The TSPX Expression Level Is Associated with the Clinical Outcomes of Lung Adenocarcinoma

**Figure 1:**
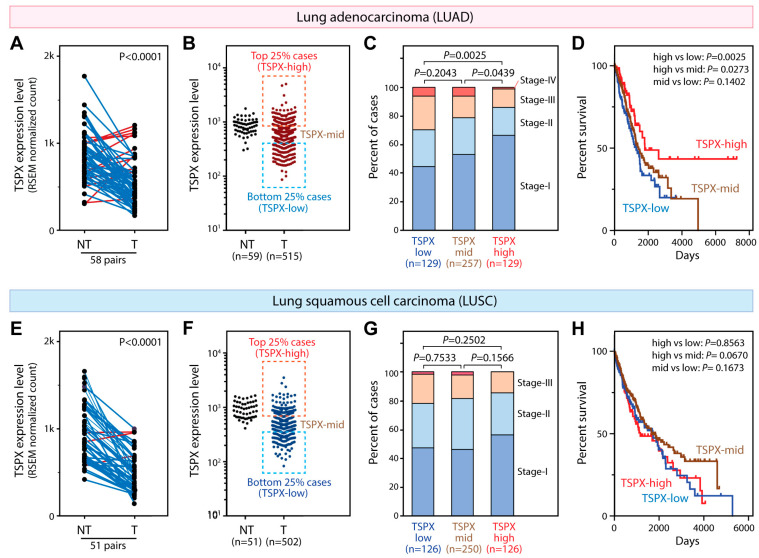
TSPX expression levels in relation to the pathologic stage and survival ratios of lung adenocarcinoma and squamous cell carcinoma patients. (**A**) TSPX expression levels in 58 lung adenocarcinoma tumor (T)/non-tumor (NT) paired samples from TCGA. Expression values (RSEM normalized count values) were plotted, with paired samples linked by a solid line; blue, decrease; red, increase. The *p*-value of the Wilcoxon matched pairs signed rank test is shown. (**B**) TSPX expression levels in 59 NT and 515 lung adenocarcinoma cases. The latter were divided into the TSPX-high group (highest 25%, *n* = 129), TSPX-low (lowest 25% cases, *n* = 129), and TSPX-mid (*n* = 257) group. (**C**) Distributions of pathologic stages (I-IV) across the TSPX-low, TSPX-mid, and TSPX-high groups. Chi-squared test *p*-value is indicated. (**D**) Survival curves for the TSPX-high (red), TSPX-mid (brown), and TSPX-low (blue) groups. Log-rank test *p*-value is indicated. (**E**) TSPX expression levels in 51 lung squamous cell carcinoma tumor/non-tumor paired samples from TCGA, similar to A. (**F**) TSPX expression levels in 51 NT and 502 lung squamous cell carcinoma cases, categorized into TSPX-high (highest 25% cases, *n* = 126), TSPX-low (lowest 25% cases, *n* = 126), and TSPX-mid (*n* = 246) groups. (**G**) Distributions of pathologic stages between the TSPX-low, TSPX-mid, and TSPX-high groups for lung squamous cell carcinoma, similar to C. Red indicates Stage-IV. Chi-squared test *p*-value is indicated. (**H**) Survival curves for the TSPX-high (red), TSPX-mid (brown), and TSPX-low (blue) groups in lung squamous cell carcinoma. Log-rank test *p*-value is indicated.

To explore the expression pattern of TSPX in lung cancer, we obtained the RNA-seq transcriptome data and clinical information of lung adenocarcinoma (*n* = 515) and lung squamous cell carcinoma (*n* = 502) samples from TCGA. Of the 58 cases with tumor and non-tumor paired lung adenocarcinoma samples, TSPX was downregulated in 47 cases (81%) as compared to the adjacent non-tumor specimens (Figure 1A), indicating that TSPX was significantly downregulated in lung adenocarcinoma (Wilcoxon matched pair test *p*-value < 0.0001). Next, we correlated the TSPX expression level in cancer with the clinical information such as pathological stages/tumor grades and patient mortality. Among the 515 lung adenocarcinoma cases, the top 25% cases (*n* = 129) expressed TSPX at the highest level and were classified as the TSPX-high group, the bottom 25% cases (*n* = 129) expressed TSPX at the lowest level and were classified as the TSPX-low group, and the rest (*n* = 257) were classified as the TSPX-mid group (Figure 1B). Our analysis showed that patients of the TSPX-high group were diagnosed at earlier pathologic/tumor stages, as compared to those of the TSPX-low group, i.e., while 66% of patients of the TSPX-high group were at stage-I, only 45% patients of the TSPX-low group were at stage-I (*p*-value = 0.0027) (Figure 1C). Patients in the TSPX-mid group at stage-I occupied an intermediate position between the TSPX-high and TSPX-low groups (Figure 1C). Similarly, the survival rate of the TSPX-high group was significantly higher than those of the TSPX-low and TSPX-mid groups (log-rank test *p*-value = 0.0025 and 0.0273, respectively) (Figure 1D). The median survival time for the TSPX-high group was 1798 days, which was 510 days longer than that of the TSPX-low group (1288 days) and 377 days longer than that of the TSPX-mid group (1421 days). Additionally, the 5-year survival ratio was 49% in the TSPX-high group, compared to 33% in the TSPX-low group and 40% in the TSPX-mid group. The log-rank hazard ratio between the TSPX-high and TSPX-low groups was 0.5171 (95% CI: 0.3404 to 0.7854), indicating a significantly lower risk in the TSPX-high group. There was no significant difference in survival rate between the TSPX-low group and TSPX-mid group (Figure 1D). These observations suggest that reduced levels of TSPX expression could be directly or indirectly associated with the progression and malignancy of lung adenocarcinoma.

Similar analyses of the transcriptome and clinical data of lung squamous cell carcinoma (LUSC) in TCGA datasets showed that TSPX was significantly downregulated in lung squamous cell carcinoma (Figure 1E). TSPX was downregulated in 48 out of 51 cases (94%) with paired tumor and non-tumor lung squamous cell carcinoma samples (Wilcoxon matched pair test *p*-value < 0.0001). In the lung squamous cell carcinoma samples, although there was a similar, but less robust, distribution trend of pathological stages from TSPX-low to TSPX-high groups as in the lung adenocarcinoma samples (Figure 1G), there was also no significant difference in survival rate between TSPX-low group and TSPX-high group (Figure 1H).

These observations suggest that the TSPX expression level could be correlated with cancer aggressiveness in lung adenocarcinoma, but not as pronounced in lung squamous cell carcinoma, making it a potential differentiating diagnostic/prognostic marker for the two subtypes of NSCLC.

### 3.2. Overexpression of TSPX Inhibits Cell Proliferation in NSCLC Cell Lines

**Figure 2:**
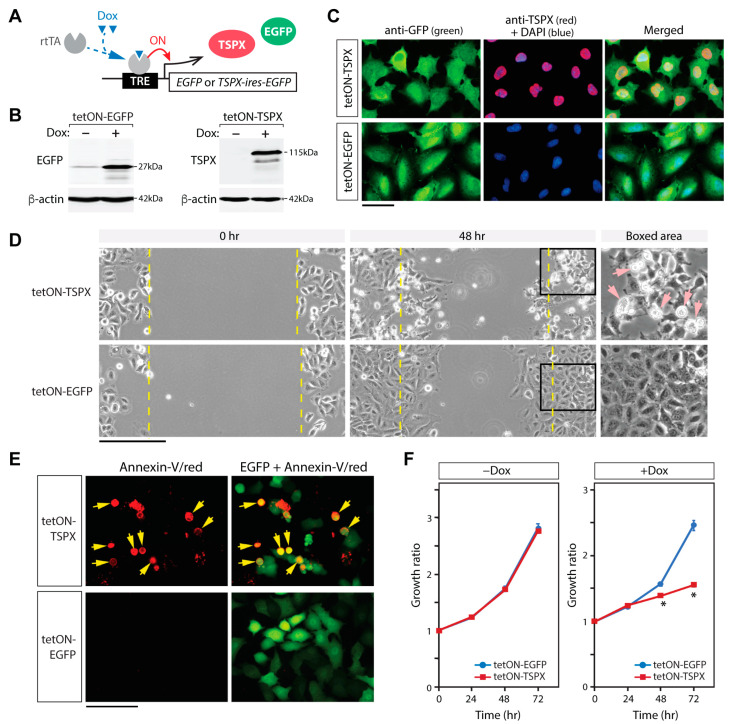
Overexpression of TSPX inhibits cell proliferation and causes cell death in A549 cells. (**A**) Diagram of the tet-ON transgene activation system. Addition of doxycycline (Dox) in the culture medium recruited the transactivator rtTA onto the tetracycline responsive (TRE) promoter and activated the target transgene. (**B**) The expressions of EGFP and TSPX in the respective transduced A549 cells with (+) and without (−) Dox induction were confirmed by Western blot, using β-actin as a reference. (**C**) Immunofluorescence of EGFP (green, left), TSPX (red, middle), and DAPI staining (blue) in the respective transduced A549 cells at 24 h after Dox induction. Far right panels show the merged images of TSPX, EGFP, and DAPI staining. (**D**) Scratch tests for A549-tetON-EGFP cells (left) and A549-tetON-TSPX cells (right) under a Dox-induction condition. Far right panel shows a magnified image of the boxed area. Yellow line indicates the wound edges at 0 h. Pink arrows indicate detached A549-tetON-TSPX cells. (**E**) Annexin-V binding assay (red) at 48 h after Dox induction showed that the detached A549-tetON-TSPX cells were positively stained by Annexin-V conjugated with Alexa Fluor 594 (red), corresponding to dead or apoptotic cells. Yellow arrows indicate cells stained with Annexin-V/Alexa Fluor 594. (**F**) Cell proliferation of A549-tetON-TSPX cells was inhibited under the Dox-induction condition (+Dox, right) but not under uninduced condition (−Dox, left), comparing with A549-tetON-EGFP cells, indicating that the TSPX overexpression inhibited cell proliferation. Asterisks indicate the significant difference at Student’s *t*-test *p*-value < 0.05. Scale bar = 50 µm in (**C**), 100 µm in (**D**,**E**).

To examine the effects of TSPX in NSCLC cells, A549 lung adenocarcinoma cells and SK-MES-1 lung squamous cell carcinoma cells were transduced with the tet-ON lentiviral vector system expressing EGFP and TSPX under control of doxycycline (Dox) (Figure 2A). The resultant cells were designated as A549-tetON-TSPX and MES1-tetON-TSPX, respectively. A549-tetON-EGFP cells and MES1-tetON-EGFP cells that expressed EGFP alone were used as references in these experiments. Western blot analysis confirmed that the expression of TSPX and EGFP were appropriately controlled by Dox treatment in the transduced cells (Figure 2B). An immunofluorescent analysis showed that the overexpressed TSPX was primarily localized in the nuclei in the A549-tetON-TSPX cells, similarly observed in other cell lines, including prostate cancer LNCaP, cervical cancer HeLa, and osteosarcoma U2OS cells (Figure 2C, red color) [16,43,44].

Scratch tests showed that the overexpression of TSPX in A549-tetON-TSPX cells resulted in a dramatic change in cell morphology, lower cell density, and slower closing/regrowth of the scratched area, as compared with the control A549-tetON-EGFP cells at 48 h after scratching (Figure 2D). Numerous cells overexpressing TSPX detached from the dish surface and floated in the culture medium (Figure 2D, boxed area). An Annexin-V binding assay, performed at 48 h after Dox-induction, showed that the most floating cells were dead/apoptotic cells positively stained by Annexin-V (Figure 2E, red-stained cells). In addition, a cell proliferation assay performed at 0, 24, 48, and 72 h demonstrated that the TSPX overexpression significantly suppressed overall cell proliferation by 48 h after Dox induction (Figure 2F).

**Figure 3:**
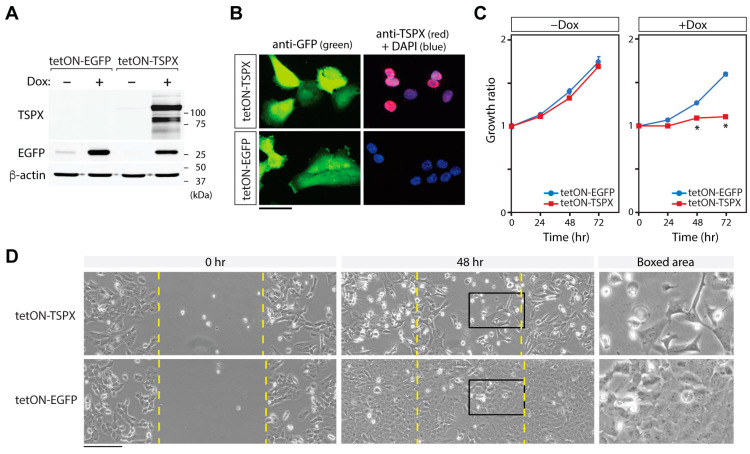
Overexpression of TSPX inhibits cell proliferation in SK-MES-1 cells. (**A**) The expressions of EGFP and TSPX in the respective transduced SK-MES-1 cells with (+) and without (−) Dox induction were confirmed by Western blot. β-actin was analyzed as a reference. (**B**) Immunofluorescence of EGFP (green), TSPX (red), and DAPI staining (blue) in the respective transduced SK-MES-1 cells at 24 h after Dox-induction. Right panels show the merged images of TSPX and DAPI staining. (**C**) Cell proliferation of MES1-tetON-TSPX cells was inhibited under the Dox-induction condition (+Dox, right) but not under uninduced condition (−Dox, left), comparing with MES1-tetON-EGFP cells, indicating that the TSPX overexpression inhibited cell proliferation in SK-MES-1 cells. Asterisks indicate the significant difference at Student’s *t*-test *p*-value < 0.05. (**D**) Scratch tests for MES1-tetON-TSPX cells (top panels) and MES1-tetON-EGFP cells (bottom panels) under a Dox-induction condition. Phase contrast images show the representative gaps at 0 h and 48 h after scratch and Dox-induction. Far right panels show digitally magnified images of the boxed areas, respectively. The scratched area was completely healed by MES1-tetON-EGFP, but not by MES1-tetON-TSPX cells at 48 h. Scale bar = 100 µm in (**B**), 200 µm in (**D**).

Similarly to A549-tetON-TSPX cells, the overexpressed TSPX induced by Dox treatment was primarily localized to the nuclei in MES1-tetON-TSPX cells (Figure 3A,B). A cell proliferation assay and scratch tests for MES1-tetON-TSPX cells showed that the overexpression of TSPX in SK-MES-1 cells also significantly suppressed cell proliferation by 48 h after Dox induction (Figure 3C) and resulted in slower healing of the scratched area (Figure 3D). These results further confirm that TSPX plays an important role in inhibiting cell proliferation and cell migration in NSCLC cells *in vitro*.

### 3.3. Identification of the TSPX Downstream Target Genes and Pathways in NSCLC Cell Lines

**Figure 4:**
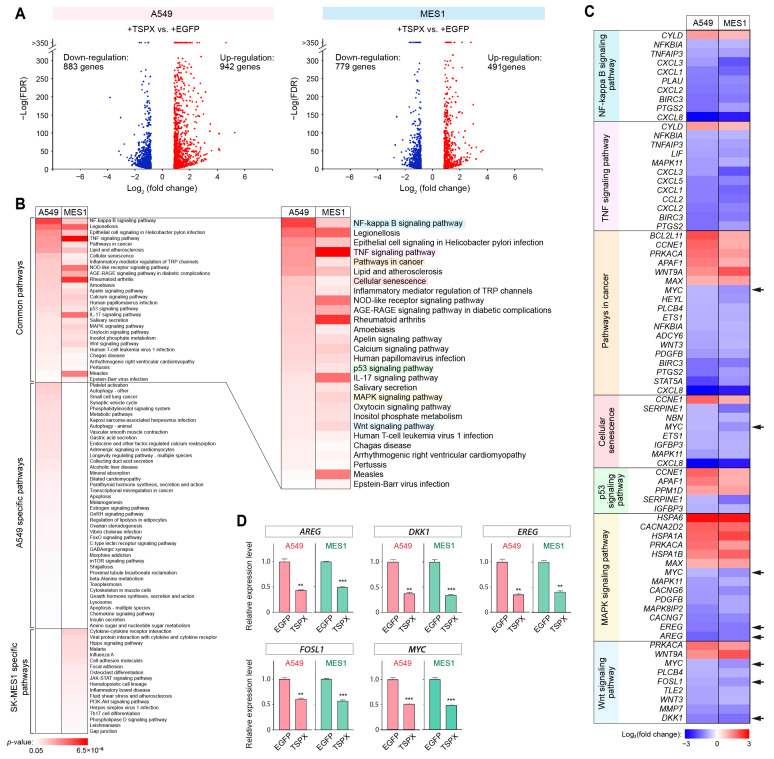
Identification of the TSPX downstream genes and their biological processes in A549 and SK-MES-1 cells. (**A**) Volcano plots representing DEGs between TSPX-overexpressing and control cells in A549 (left) and SK-MES-1 (right, abbreviated as MES1) cell lines. The list of DEGs is shown in Supplementary Table S1. (**B**) Results of the DAVID pathway enrichment analysis for DEGs in TSPX-overexpressing A549 and SK-MES-1 cells. The pathways that were commonly enriched in both A549 and SK-MES-1 cells are shown on the right. A magnified image of all enriched pathways, including the cell line-specific pathways, is shown in Supplementary Figure S1. (**C**) Gene expression changes in DEGs involved in the selected pathways in (**B**). A549, DEGs in TSPX-overexpressing A549 cells; MES1, DEGs in TSPX-overexpressing SK-MES-1 cells. Only DEGs shared between A549 and SK-MES-1 cells are shown. Arrows indicate the genes analyzed in (**D**). (**D**) Validation of the gene expression changes induced by TSPX overexpression in A549 cells and SK-MES-1 cells by quantitative RT-PCR. Values represent the relative expression levels of AREG, DKK1, EREG, FOSL1, and MYC in A549-tetON-TSPX cells or MES1-tetON-TSPX cells (TSPX) at 24 h after Dox induction (mean ± standard error, *n* = 3). A549-tetON-EGFP cells and MES1-tetON-EGFP cells were used as references (EGFP), respectively. Values were normalized against GAPDH as an internal control. **, Student’s *t*-test *p*-value ≤ 0.001; ***, *p*-value ≤ 0.0001.

To investigate the TSPX-mediated mechanisms underlying the suppression of cell proliferation and cell migration through gene regulation, we identified the downstream genes and pathways modulated by TSPX in NSCLC cells using RNA-seq transcriptome analyses. Initially, RNA-seq transcriptome analysis was performed in biological triplicates on A549-tetON-TSPX cells and MES1-tetON-TSPX cells, with A549-tetON-EGFP cells and MES1-tetON-EGFP cells serving as controls, respectively, using the Illumina RNA-seq platform. mRNAs were isolated from these cells after Dox induction for 24 h and analyzed to determine the changes resulting from TSPX overexpression. Genes representing changes with TCC/edgeR software analysis FDR < 0.005, Student’s *t*-test *p*-value < 0.05, Log_2_(gene expression level) > 4, and |Log_2_(fold change)| > 0.85 were considered as differentially expressed genes (DEGs). The results showed that 1825 genes, including 942 upregulated genes and 883 downregulated genes, were differentially expressed by TSPX overexpression in A549 cells (Figure 4A left panel and Table S2A), while 1270 genes, including 491 upregulated genes and 779 downregulated genes, were differentially expressed in SK-MES-1 cells (Figure 4A right panel and Table S2B).

To gain insights into the biological processes mediated by TSPX, the differentially regulated genes (DEGs) were analyzed for pathway enrichment using the DAVID bioinformatics resources [32] for A549 and SK-MES-1 cells, respectively. The results showed that, while many cell-type specific pathways were enriched in each cell line, the pathways associated with cell proliferation and cell viability, including NF-kappa B, TNF, cellular senescence, p53, MAPK, and Wnt signaling pathways, were commonly and significantly enriched in both cell lines (Figure 4B and Supplementary Figure S1). Further, TSPX consistently regulated the genes involved in these pathways in the same direction, either up- or downregulation, in both A549 and SK-MES-1 cells (Figure 4C).

To validate the results of the RNA-seq analysis, quantitative RT-PCR analysis was performed on five selected genes, *AREG*, *EREG*, *FOSL1*, *DKK1,* and *MYC*, which were closely correlated with lung cancer development and were downregulated by TSPX-overexpression in both A549 and SK-MES-1 cells (Figure 4C, arrows) [45,46,47,48,49,50,51,52,53,54,55]. The results showed that these five genes were significantly suppressed by TSPX overexpression in both A549 and SK-MES-1 cells, thereby confirming the RNA-seq data (Figure 4D).

### 3.4. Identification of the Potential Target Genes of TSPX in Clinical Samples of Lung Adenocarcinoma

**Figure 5:**
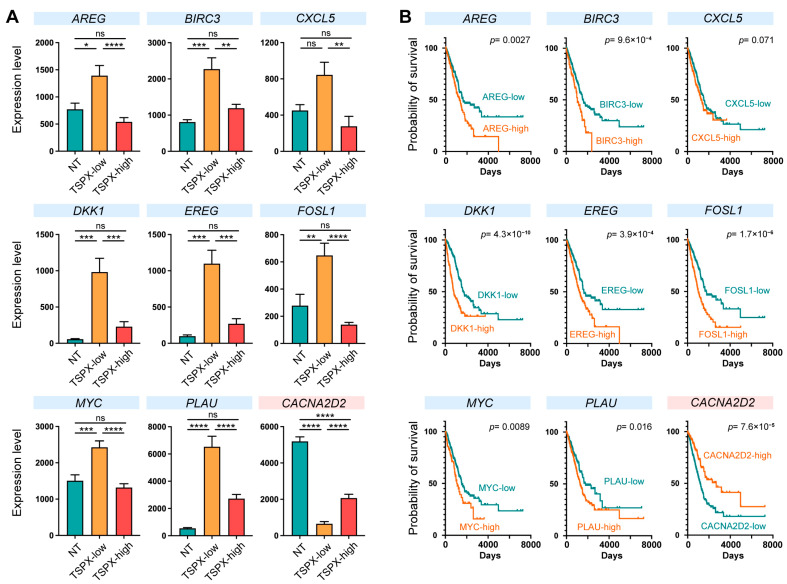
Gene expression patterns of TSPX-downstream genes in clinical lung adenocarcinoma from TCGA datasets. (**A**) Expression levels (RSEM normalized read counts) of *AREG*, *BIRC3*, *CXCL5*, *DKK1*, *EREG*, *FOSL1*, *MYC*, *PLAU*, and *CACNA2D2*, in non-tumor lung tissues (NT), TSPX-low lung adenocarcinoma group (TSPX-low), and TSPX-high lung adenocarcinoma group (TSPX-high) are shown. Statistical significance was determined by one-way ANOVA with Tukey’s multiple comparison test; * *p* ≤ 0.05; ** *p* ≤ 0.01; *** *p* ≤ 0.001; **** *p* ≤ 0.0001; ns, not significant. Error bars represent mean ± SEM. (**B**) Correlation between the expression levels of the indicated genes and patient survival. Survival curves for high expressors (orange) or low expressors (green) are shown. Log-rank test *p*-values were obtained from TCGA datasets via the Human Protein Atlas (HPA).

As shown in Figure 1, a high expression level of TSPX correlated with better prognosis and survival in lung adenocarcinoma patients. To correlate the experimental data with in silico data, the transcriptomes of lung adenocarcinomas from the TCGA database with high TSPX expression were compared with transcriptomes with low TSPX expression [31]. The results showed that, among the 45 TSPX-mediated DEGs involved in pathways related to cell proliferation and cell viability in A549 and SK-MES-1 cells (Figure 4C), nine genes showed the same expression pattern in clinical samples of lung adenocarcinomas in relation to TSPX expression level, i.e., AREG, *BIRC3*, *CXCL5*, *DKK1*, *EREG*, *FOSL1*, *MYC*, *PLAU*, and *CACNA2D2* (Figure 5A). While *CACNA2D2* was upregulated by TSPX, the remaining eight genes were downregulated by TSPX (Figure 5A). The expression levels of these genes were significantly altered in the TSPX-low group, whereas they were more or less unaltered in the TSPX-high group (Figure 5A).

To further identify the TSPX downstream genes potentially playing important roles related to clinical outcome, these nine genes were analyzed in correlation between their expression patterns and the mortality of lung adenocarcinoma patients by the Human Protein Atlas portal (HPA) [42]. The results showed that the expression levels of *AREG*, *BIRC3*, *DKK1*, *EREG*, *FOSL1*, *MYC*, and *PLAU* were negatively correlated with survival rate, while that of *CACNA2D2* was positively correlated with better survival rate (Figure 5B). The expression level of *CXCL5* was not correlated with patient survival in the TCGA lung adenocarcinoma database. Furthermore, the log-rank hazard ratios from survival analyses were as follows: *AREG*, 1.549 (95% CI: 1.154–2.078); BIRC3, 1.728 (95% CI: 1.171–2.550); CXCL5, 1.319 (95% CI: 0.960–1.814); DKK1, 2.445 (95% CI: 1.746–3.425); EREG, 1.687 (95% CI: 1.260–2.259); FOSL1, 2.002 (95% CI: 1.468–2.732); MYC, 1.541 (95% CI: 1.067–2.228); PLAU, 1.455 (95% CI: 1.084–1.952); and CACN2D2 = 0.501 (95% CI: 0.374–0.672).

Taken together, our results suggest that TSPX may suppress the development and/or progression of lung adenocarcinoma by downregulating *AREG*, *BIRC3*, *DKK1*, *EREG*, *FOSL1*, *MYC*, and *PLAU*, which could exacerbate lung adenocarcinoma, and upregulating the tumor suppressor *CACNA2D2*.

## 4. Discussion

TSPX was initially identified as a TGF-β-inducible gene in non-small cell lung cancer (NSCLC) cell lines [10]. A subsequent study demonstrated that TSPX could upregulate p21 (Waf1/Cip1) and inhibit cell proliferation in both p53-dependent and -independent manners in cultured NSCLC cell lines; therefore, it has been considered as a tumor suppressor for lung cancer [27]. Furthermore, it was also demonstrated that TSPX could suppress proto-oncogene TrkC (also known as NTRK3) by forming TSPX/REST complex on the target gene promoter and/or activate p53 protein via the SIRT1-p300 pathway [26,30]. In the present study, using RNA-seq transcriptome analyses of the TSPX-overexpressing A549 and SK-MES-1 cells and datamining of TCGA LUAD data, we demonstrated that TSPX could regulate numerous genes involved in various oncogenic processes (Figure 4C). In particular, we identified eight potential TSPX downstream genes, i.e., AREG, *BIRC3*, *DKK1*, *EREG*, *FOSL1*, *MYC*, *PLAU*, and *CACNA2D2*, whose altered expressions contribute to cell proliferation/viability and clinical outcomes in LUAD (Figure 5). Importantly, their respective differential expression patterns were correlated between the A549 cell model and TCGA LUAD transcriptomes, supporting the validity of our TSPX overexpression analysis in LUAD cell system.

**Figure 6:**
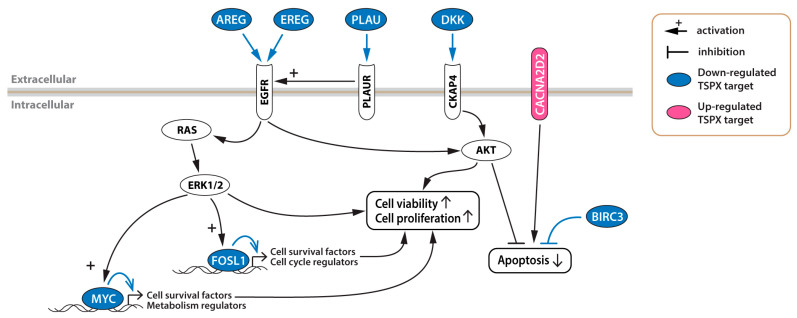
Schematic diagram illustrating the functions of the identified TSPX-downstream genes in the EGFP-signaling and other pathways involved in cell survival in lung adenocarcinoma. Details of respective functions are described in the body text.

Among the eight differentially expressed genes, five have been demonstrated to be involved in the EGFR signaling pathway. Specifically, the *AREG* and *EREG* genes encode the EGFR ligand amphiregulin and epiregulin, respectively. Amphiregulin is significantly upregulated in advanced NSCLC patients with poor response to Gefitinib. Various *in vivo* and *in vitro* studies demonstrated that amphiregulin exacerbates lung cancer cell proliferation and tumor growth by stimulating the EGFR family members to activate their downstream pathways [49,56,57]. Similarly, overexpressed EREG in NSCLC contributes to cancer cell proliferation and drug resistance [58,59]. Interestingly, treatment with EGFR tyrosine kinase inhibitor (EGFR-TKI) reduced the EREG expression level in EGFR-mutant NSCLC cells, and EREG knockdown resulted in the inhibition of cell proliferation and invasion, suggesting that EREG plays an important oncogenic role in NSCLC progression [60]. FOSL1 and MYC are oncogenic transcription factors that contribute to the development and progression of various cancer types, including lung cancer, and their expression levels have been shown to be upregulated by the EGFR-ERK1/2 signaling pathway (Figure 6) [45,46,47,48,49,50,51]. MYC is upregulated in 78% of NSCLC cases, and MYC gene amplification is detected in approximately 8% of NSCLC cases, highlighting the significant contribution of MYC in NSCLC development [45,61]. High FOSL1 expression is associated with poor survival outcomes, likely due to its role in upregulating the AURKA gene, which encodes the cell division regulator aurora kinase A [48]. Notably, FOSL1 is a key regulator of the AREG gene, and aberrant activation of KRAS could potentiate the EGFR signaling through the EGFR-KRAS-FOSL1-AREG positive feedback loop [49]. PLAU (also known as urokinase-type plasminogen activator (uPA)) is a ligand of the plasminogen activator, urokinase receptor (PLAUR, also known as uPAR). The PLAU-PLAUR system is involved in cancer progression and metastasis in various types of cancer [62]. In particular, in NSCLC, PLAU-PLAUR interacts with EGFR and confers cancer cell proliferation and gefitinib resistance by activating the EGFR-ERK1/2 signaling pathway and AKT-survivin pathway [63,64,65].

The expression levels of *AREG*, *BIRC3*, *DKK1*, *EREG*, *FOSL1*, *MYC*, and *PLAU* are negatively correlated with the survival ratio, and are significantly higher in the TSPX-low lung adenocarcinoma samples, compared to TSPX-high lung adenocarcinoma samples (Figure 5). Since TSPX overexpression significantly suppressed these genes in cultured NSCLC cells (Figure 4), we propose that TSPX suppresses these genes to inhibit the initiation and progression of lung adenocarcinoma under clinical conditions. Importantly, *AREG*, *EREG*, *FOSL1*, *MYC*, and *PLAU* are involved in the EGFR signaling pathway (Figure 6). Abnormal activation of EGFR plays a central role in the development of NSCLC [2,3]. The activated EGFR stimulates various downstream signaling pathways, including RAS-RAF-MEK-ERK and PI3K-Akt-mTOR pathways, that promote cell proliferation and inhibit cell death [66,67]. Collectively, our findings suggest that pathologic downregulation of TSPX in NSCLC, especially in lung adenocarcinoma, results in upregulation of *AREG*, *EREG*, *FOSL1*, *MYC*, and *PLAU*, thereby potentiating the EGFR signaling pathway and leading to the initiation and/or exacerbation of cancer development (Figure 6).

BIRC3 is a member of the inhibitors of apoptosis proteins (IAPs) that contributes to therapeutic resistance in NSCLC and glioblastoma [68,69]. DKK1 is a secreted protein that is frequently upregulated in NSCLC and involved in invasive growth of NSCLC by activating the β-catenin signaling pathway [52,53]. Recent studies have demonstrated a novel pathway where DKK1 binds to its novel receptor CKAP4 and activates the Akt-signaling pathway for cancer progression in lung cancer, pancreatic cancer, and esophageal cancer [70,71].

On the other hand, our study showed that TSPX upregulates the tumor suppressor gene *CACNA2D2* in NSCLC cells (Figure 4). The level of *CACNA2D2* is significantly higher in the TSPX-high lung adenocarcinoma samples, as compared with TSPX-low lung adenocarcinoma samples (Figure 5). CACNA2D2 is a subunit of the Ca^2+^ channel complex that was initially identified as a tumor suppressor gene located in the lung cancer homozygous deletion region of chromosome 3p21.3 [72]. Overexpression of CACNA2D2 in NSCLC cells inhibited cell proliferation and invasion, and induced apoptosis by regulating intracellular Ca^2+^ signaling and/or homeostasis [73,74]. The upregulation of CACNA2D2 by TSPX may reinforce the inhibition of cancer development.

In contrast to lung adenocarcinoma, survival analysis of the TCGA lung squamous cell carcinoma (LUSC) dataset revealed that the expression levels of *BIRC3*, *CXCL5*, *DKK1*, *FOSL1*, and *MYC* exhibited limited association with patient survival (Supplementary Figure S2). Notably, *DKK1* and *MYC* were not associated with survival in lung squamous cell carcinoma patients, despite their high expression being significantly correlated with poor survival outcomes in lung adenocarcinoma patients. Furthermore, high expression of *CACNA2D2* was correlated with poor survival in lung squamous cell carcinoma patients, contrasting with findings in lung adenocarcinoma. Since lung adenocarcinoma and squamous cell carcinoma, subtypes of NSCLC, originate from different cell types and exhibit different dependencies on the EGFR signaling pathway, with lung adenocarcinoma relying more strongly [75,76,77], these results suggest that TSPX-regulated genes, particularly those of the EGFR signaling pathway, may have lesser or different roles in lung squamous cell carcinoma pathogenesis under clinical conditions compared to that of lung adenocarcinoma.

At present, the mechanisms by which TSPX regulates its downstream genes in cancer are currently not fully identified. Recent studies suggest that aberrant DNA hypermethylation could be a potential contribution factor [15,18]. TSPX harbors a SET/NAP domain, that plays an important role in chromatin structure, histone modification, and gene regulation [21,22,23]. Hence, TSPX may exert its transcriptional regulation functions via direct interactions on its target genes. Indeed, it was reported that TSPX is an essential component of the REST/HDAC repressor complex that is involved in transcriptional suppression of the target genes [26]. Previously, we demonstrated that TSPX could bind androgen receptor (AR) and inhibit its transactivation function [29]. Furthermore, we demonstrated that TSPX binds directly to the promoter region of the *MYC* gene and suppresses the *MYC* expression in prostate cancer LNCaP cells [16]. The TSPX-mediated suppression of the MYC gene was consistently observed in A549 and SK-MES-1 cells in the present study (Figure 4). Future studies on the mechanisms of TSPX-mediated gene regulation, as well as those regulating its own expression, will provide novel clues to understand its tumor suppressor functions.

The present study used established cell lines in a 2D culture system, which may not fully replicate the complexity of *in vivo* tumor microenvironments. Our results, however, have provided the initial clues in establishing the potential involvement of TSPX in the EGFR signaling and oncogenesis in lung adenocarcinoma, and a rationale for more detailed analyses with advanced strategies, such as *in vivo* CRISPR gene activation/inactivation [78,79], of this X-linked tumor suppressor gene in both preclinical and clinical models [80,81]. These additional studies could offer more physiological relevance, thereby revealing critical insights into the relationship between TSPX expression levels and clinical pathogenesis/outcomes in lung cancer.

## 5. Conclusions

Non-small cell lung cancer (NSCLC) is a highly heterogeneous and aggressive cancer, displaying significant variability in progression and response to treatments among patients [2,5,82,83]. Our study established (1) the effects of the tumor suppressor TSPX on the EGFR signaling oncogenic pathway, among others, in lung adenocarcinoma pathogenesis and clinical outcomes and (2) highlighted TSPX as a potential marker for differential diagnosis and prognosis of the two subtypes, lung adenocarcinoma and squamous cell carcinoma, of NSCLC.

## Data Availability

The original contributions presented in the study are included in the article, further inquiries can be directed to the corresponding author.

## Supplementary Materials

The following supporting information can be downloaded at: https://www.mdpi.com/article/10.3390/genes16010075/s1, Table S1: Primer sequences for quantitative RT-PCR analyses. Table S2: (A) Differentially expressed genes in A549-tetON-TSPX cells compared to A549-tetON-EGFP cells. (B) Differentially expressed genes in MES1-tetON-TSPX cells compared to MES1-tetON-EGFP cells. Figure S1: Results of DAVID pathway enrichment analysis for differentially expressed genes in TSPX-overexpressing A549 and SK-MES-1 cells. Figure S2: Correlations between the expression levels of TSPX downstream genes and patient survival in the TCGA lung squamous cell carcinoma dataset.

## References

[1] Bray F., Ferlay J., Soerjomataram I., Siegel R.L., Torre L.A., Jemal A. (2018). Global cancer statistics 2018: GLOBOCAN estimates of incidence and mortality worldwide for 36 cancers in 185 countries. CA Cancer J. Clin..

[2] Herbst R.S., Morgensztern D., Boshoff C. (2018). The biology and management of non-small cell lung cancer. Nature.

[3] Reck M., Heigener D.F., Mok T., Soria J.C., Rabe K.F. (2013). Management of non-small-cell lung cancer: Recent developments. Lancet.

[4] Miller K.D., Siegel R.L., Lin C.C., Mariotto A.B., Kramer J.L., Rowland J.H., Stein K.D., Alteri R., Jemal A. (2016). Cancer treatment and survivorship statistics, 2016. CA Cancer J. Clin..

[5] Chen Z., Fillmore C.M., Hammerman P.S., Kim C.F., Wong K.K. (2014). Non-small-cell lung cancers: A heterogeneous set of diseases. Nat. Rev. Cancer.

[6] Wang D.C., Wang W., Zhu B., Wang X. (2018). Lung Cancer Heterogeneity and New Strategies for Drug Therapy. Annu. Rev. Pharmacol. Toxicol..

[7] Thai A.A., Solomon B.J., Sequist L.V., Gainor J.F., Heist R.S. (2021). Lung cancer. Lancet.

[8] Nair N.U., Greninger P., Zhang X., Friedman A.A., Amzallag A., Cortez E., Sahu A.D., Lee J.S., Dastur A., Egan R.K. (2023). A landscape of response to drug combinations in non-small cell lung cancer. Nat. Commun..

[9] Vargas A.J., Harris C.C. (2016). Biomarker development in the precision medicine era: Lung cancer as a case study. Nat. Rev. Cancer.

[10] Ozbun L.L., You L., Kiang S., Angdisen J., Martinez A., Jakowlew S.B. (2001). Identification of differentially expressed nucleolar TGF-beta1 target (DENTT) in human lung cancer cells that is a new member of the TSPY/SET/NAP-1 superfamily. Genomics.

[11] Martinez A., Ozbun L.L., Angdisen J., Jakowlew S.B. (2002). Expression of differentially expressed nucleolar transforming growth factor-beta1 target (DENTT) in adult mouse tissues. Dev. Dyn..

[12] Consortium G.T. (2013). The Genotype-Tissue Expression (GTEx) project. Nat. Genet..

[13] Kido T., Lo R.C., Li Y., Lee J., Tabatabai Z.L., Ng I.O., Lau Y.F. (2014). The potential contributions of a Y-located protooncogene and its X homologue in sexual dimorphisms in hepatocellular carcinoma. Hum. Pathol..

[14] Eyler C.E., Wu Q., Yan K., MacSwords J.M., Chandler-Militello D., Misuraca K.L., Lathia J.D., Forrester M.T., Lee J., Stamler J.S. (2011). Glioma stem cell proliferation and tumor growth are promoted by nitric oxide synthase-2. Cell.

[15] Kandalaft L.E., Zudaire E., Portal-Nunez S., Cuttitta F., Jakowlew S.B. (2008). Differentially expressed nucleolar transforming growth factor-beta1 target (DENTT) exhibits an inhibitory role on tumorigenesis. Carcinogenesis.

[16] Kido T., Li Y., Tanaka Y., Dahiya R., Lau Y.F. (2019). The X-linked tumor suppressor TSPX downregulates cancer-drivers/oncogenes in prostate cancer in a C-terminal acidic domain dependent manner. Oncotarget.

[17] Zhang X., Wu X., Yao W., Wang Y.H. (2023). A tumor-suppressing role of TSPYL2 in thyroid cancer: Through interacting with SIRT1 and repressing SIRT1/AKT pathway. Exp. Cell Res..

[18] Kim T.Y., Zhong S., Fields C.R., Kim J.H., Robertson K.D. (2006). Epigenomic profiling reveals novel and frequent targets of aberrant DNA methylation-mediated silencing in malignant glioma. Cancer Res..

[19] Le Gallo M., O’Hara A.J., Rudd M.L., Urick M.E., Hansen N.F., O’Neil N.J., Price J.C., Zhang S., England B.M., Godwin A.K. (2012). Exome sequencing of serous endometrial tumors identifies recurrent somatic mutations in chromatin-remodeling and ubiquitin ligase complex genes. Nat. Genet..

[20] Sato S., Maekawa R., Yamagata Y., Asada H., Tamura I., Lee L., Okada M., Tamura H., Sugino N. (2014). Potential mechanisms of aberrant DNA hypomethylation on the x chromosome in uterine leiomyomas. J. Reprod. Dev..

[21] Gamble M.J., Erdjument-Bromage H., Tempst P., Freedman L.P., Fisher R.P. (2005). The histone chaperone TAF-I/SET/INHAT is required for transcription in vitro of chromatin templates. Mol. Cell. Biol..

[22] Almeida L.O., Neto M.P.C., Sousa L.O., Tannous M.A., Curti C., Leopoldino A.M. (2017). SET oncoprotein accumulation regulates transcription through DNA demethylation and histone hypoacetylation. Oncotarget.

[23] Muto S., Senda M., Akai Y., Sato L., Suzuki T., Nagai R., Senda T., Horikoshi M. (2007). Relationship between the structure of SET/TAF-Ibeta/INHAT and its histone chaperone activity. Proc. Natl. Acad. Sci. USA.

[24] Canela N., Rodriguez-Vilarrupla A., Estanyol J.M., Diaz C., Pujol M.J., Agell N., Bachs O. (2003). The SET protein regulates G2/M transition by modulating cyclin B-cyclin-dependent kinase 1 activity. J. Biol. Chem..

[25] Wang D., Kon N., Lasso G., Jiang L., Leng W., Zhu W.G., Qin J., Honig B., Gu W. (2016). Acetylation-regulated interaction between p53 and SET reveals a widespread regulatory mode. Nature.

[26] Epping M.T., Lunardi A., Nachmani D., Castillo-Martin M., Thin T.H., Cordon-Cardo C., Pandolfi P.P. (2015). TSPYL2 is an essential component of the REST/NRSF transcriptional complex for TGFbeta signaling activation. Cell Death Differ..

[27] Tu Y., Wu W., Wu T., Cao Z., Wilkins R., Toh B.H., Cooper M.E., Chai Z. (2007). Antiproliferative autoantigen CDA1 transcriptionally up-regulates p21(Waf1/Cip1) by activating p53 and MEK/ERK1/2 MAPK pathways. J. Biol. Chem..

[28] Li Y., Lau Y.F. (2008). TSPY and its X-encoded homologue interact with cyclin B but exert contrasting functions on cyclin-dependent kinase 1 activities. Oncogene.

[29] Li Y., Zhang D.J., Qiu Y., Kido T., Lau Y.C. (2017). The Y-located proto-oncogene TSPY exacerbates and its X-homologue TSPX inhibits transactivation functions of androgen receptor and its constitutively active variants. Hum. Mol. Genet..

[30] Magni M., Buscemi G., Maita L., Peng L., Chan S.Y., Montecucco A., Delia D., Zannini L. (2019). TSPYL2 is a novel regulator of SIRT1 and p300 activity in response to DNA damage. Cell Death Differ..

[31] Cancer Genome Atlas Research N., Weinstein J.N., Collisson E.A., Mills G.B., Shaw K.R., Ozenberger B.A., Ellrott K., Shmulevich I., Sander C., Stuart J.M. (2013). The Cancer Genome Atlas Pan-Cancer analysis project. Nat. Genet..

[32] Sherman B.T., Hao M., Qiu J., Jiao X., Baseler M.W., Lane H.C., Imamichi T., Chang W. (2022). DAVID: A web server for functional enrichment analysis and functional annotation of gene lists (2021 update). Nucleic Acids Res..

[33] Edwards A., Civitello A., Hammond H.A., Caskey C.T. (1991). DNA typing and genetic mapping with trimeric and tetrameric tandem repeats. Am. J. Hum. Genet..

[34] Cory G. (2011). Scratch-wound assay. Methods Mol. Biol..

[35] Kido T., Ou J.H., Lau Y.F. (2011). The X-linked tumor suppressor TSPX interacts and promotes degradation of the hepatitis B viral protein HBx via the proteasome pathway. PLoS ONE.

[36] Andrews S. FastQC: A Quality Control Tool for High Throughput Sequence Data. http://www.bioinformatics.babraham.ac.uk/projects/fastqc/.

[37] Trapnell C., Pachter L., Salzberg S.L. (2009). TopHat: Discovering splice junctions with RNA-Seq. Bioinformatics.

[38] Liao Y., Smyth G.K., Shi W. (2014). featureCounts: An efficient general purpose program for assigning sequence reads to genomic features. Bioinformatics.

[39] Sun J., Nishiyama T., Shimizu K., Kadota K. (2013). TCC: An R package for comparing tag count data with robust normalization strategies. BMC Bioinformatics.

[40] Goldman M.J., Craft B., Hastie M., Repecka K., McDade F., Kamath A., Banerjee A., Luo Y., Rogers D., Brooks A.N. (2020). Visualizing and interpreting cancer genomics data via the Xena platform. Nat. Biotechnol..

[41] Li B., Dewey C.N. (2011). RSEM: Accurate transcript quantification from RNA-Seq data with or without a reference genome. BMC Bioinformatics.

[42] Uhlen M., Zhang C., Lee S., Sjostedt E., Fagerberg L., Bidkhori G., Benfeitas R., Arif M., Liu Z., Edfors F. (2017). A pathology atlas of the human cancer transcriptome. Science.

[43] Chai Z., Wu T., Dai A., Huynh P., Koentgen F., Krippner G., Ren S., Cooper M.E. (2019). Targeting the CDA1/CDA1BP1 Axis Retards Renal Fibrosis in Experimental Diabetic Nephropathy. Diabetes.

[44] Cardano M., Magni M., Alfieri R., Chan S.Y., Sabbioneda S., Buscemi G., Zannini L. (2023). Sex specific regulation of TSPY-Like 2 in the DNA damage response of cancer cells. Cell Death Dis..

[45] Dhanasekaran R., Deutzmann A., Mahauad-Fernandez W.D., Hansen A.S., Gouw A.M., Felsher D.W. (2022). The MYC oncogene—The grand orchestrator of cancer growth and immune evasion. Nat. Rev. Clin. Oncol..

[46] Hall Z., Wilson C.H., Burkhart D.L., Ashmore T., Evan G.I., Griffin J.L. (2020). Myc linked to dysregulation of cholesterol transport and storage in nonsmall cell lung cancer. J. Lipid Res..

[47] Zhu L., Chen Z., Zang H., Fan S., Gu J., Zhang G., Sun K.D., Wang Q., He Y., Owonikoko T.K. (2021). Targeting c-Myc to Overcome Acquired Resistance of EGFR Mutant NSCLC Cells to the Third-Generation EGFR Tyrosine Kinase Inhibitor, Osimertinib. Cancer Res..

[48] Vallejo A., Perurena N., Guruceaga E., Mazur P.K., Martinez-Canarias S., Zandueta C., Valencia K., Arricibita A., Gwinn D., Sayles L.C. (2017). An integrative approach unveils FOSL1 as an oncogene vulnerability in KRAS-driven lung and pancreatic cancer. Nat. Commun..

[49] Elangovan I.M., Vaz M., Tamatam C.R., Potteti H.R., Reddy N.M., Reddy S.P. (2018). FOSL1 Promotes Kras-induced Lung Cancer through Amphiregulin and Cell Survival Gene Regulation. Am. J. Respir. Cell Mol. Biol..

[50] Sobolev V.V., Khashukoeva A.Z., Evina O.E., Geppe N.A., Chebysheva S.N., Korsunskaya I.M., Tchepourina E., Mezentsev A. (2022). Role of the Transcription Factor FOSL1 in Organ Development and Tumorigenesis. Int. J. Mol. Sci..

[51] Jiang X., Xie H., Dou Y., Yuan J., Zeng D., Xiao S. (2020). Expression and function of FRA1 protein in tumors. Mol. Biol. Rep..

[52] Li S., Qin X., Guo X., Cui A., He Y., Wei S., Wang X., Shan B. (2013). Dickkopf-1 is oncogenic and involved in invasive growth in non small cell lung cancer. PLoS ONE.

[53] Zhang J., Zhang X., Zhao X., Jiang M., Gu M., Wang Z., Yue W. (2017). DKK1 promotes migration and invasion of non-small cell lung cancer via β-catenin signaling pathway. Tumour Biol..

[54] Hsu Y.L., Huang M.S., Cheng D.E., Hung J.Y., Yang C.J., Chou S.H., Kuo P.L. (2011). Lung tumor-associated dendritic cell-derived amphiregulin increased cancer progression. J. Immunol..

[55] Sunaga N., Kaira K. (2015). Epiregulin as a therapeutic target in non-small-cell lung cancer. Lung Cancer.

[56] Busser B., Sancey L., Brambilla E., Coll J.L., Hurbin A. (2011). The multiple roles of amphiregulin in human cancer. Biochim. Biophys. Acta.

[57] Ishikawa N., Daigo Y., Takano A., Taniwaki M., Kato T., Hayama S., Murakami H., Takeshima Y., Inai K., Nishimura H. (2005). Increases of amphiregulin and transforming growth factor-α in serum as predictors of poor response to gefitinib among patients with advanced non-small cell lung cancers. Cancer Res..

[58] Sunaga N., Miura Y., Masuda T., Sakurai R. (2024). Role of Epiregulin in Lung Tumorigenesis and Therapeutic Resistance. Cancers.

[59] Riese D.J., Cullum R.L. (2014). Epiregulin: Roles in normal physiology and cancer. Semin. Cell Dev. Biol..

[60] Zhang J., Iwanaga K., Choi K.C., Wislez M., Raso M.G., Wei W., Wistuba I.I., Kurie J.M. (2008). Intratumoral epiregulin is a marker of advanced disease in non-small cell lung cancer patients and confers invasive properties on EGFR-mutant cells. Cancer Prev. Res..

[61] Xu X., Sun P.L., Li J.Z., Jheon S., Lee C.T., Chung J.H. (2011). Aberrant Wnt1/β-catenin expression is an independent poor prognostic marker of non-small cell lung cancer after surgery. J. Thorac. Oncol..

[62] Masucci M.T., Minopoli M., Di Carluccio G., Motti M.L., Carriero M.V. (2022). Therapeutic Strategies Targeting Urokinase and Its Receptor in Cancer. Cancers.

[63] Eden G., Archinti M., Arnaudova R., Andreotti G., Motta A., Furlan F., Citro V., Cubellis M.V., Degryse B. (2018). D2A sequence of the urokinase receptor induces cell growth through alphavbeta3 integrin and EGFR. Cell. Mol. Life Sci..

[64] Guerrero J., Santibanez J.F., Gonzalez A., Martinez J. (2004). EGF receptor transactivation by urokinase receptor stimulus through a mechanism involving Src and matrix metalloproteinases. Exp. Cell Res..

[65] Zhou J., Kwak K.J., Wu Z., Yang D., Li J., Chang M., Song Y., Zeng H., Lee L.J., Hu J. (2018). PLAUR Confers Resistance to Gefitinib Through EGFR/P-AKT/Survivin Signaling Pathway. Cell. Physiol. Biochem..

[66] Liu X., Wang P., Zhang C., Ma Z. (2017). Epidermal growth factor receptor (EGFR): A rising star in the era of precision medicine of lung cancer. Oncotarget.

[67] Wee P., Wang Z. (2017). Epidermal Growth Factor Receptor Cell Proliferation Signaling Pathways. Cancers.

[68] Liu C., Chen Z., Ding X., Qiao Y., Li B. (2022). Ubiquitin-specific protease 35 (USP35) mediates cisplatin-induced apoptosis by stabilizing BIRC3 in non-small cell lung cancer. Lab. Investig..

[69] Wang D., Berglund A., Kenchappa R.S., Forsyth P.A., Mule J.J., Etame A.B. (2016). BIRC3 is a novel driver of therapeutic resistance in Glioblastoma. Sci. Rep..

[70] Kimura H., Fumoto K., Shojima K., Nojima S., Osugi Y., Tomihara H., Eguchi H., Shintani Y., Endo H., Inoue M. (2016). CKAP4 is a Dickkopf1 receptor and is involved in tumor progression. J. Clin. Investig..

[71] Shinno N., Kimura H., Sada R., Takiguchi S., Mori M., Fumoto K., Doki Y., Kikuchi A. (2018). Activation of the Dickkopf1-CKAP4 pathway is associated with poor prognosis of esophageal cancer and anti-CKAP4 antibody may be a new therapeutic drug. Oncogene.

[72] Lerman M.I., Minna J.D. (2000). The 630-kb lung cancer homozygous deletion region on human chromosome 3p21.3: Identification and evaluation of the resident candidate tumor suppressor genes. The International Lung Cancer Chromosome 3p21.3 Tumor Suppressor Gene Consortium. Cancer Res..

[73] Carboni G.L., Gao B., Nishizaki M., Xu K., Minna J.D., Roth J.A., Ji L. (2003). CACNA2D2-mediated apoptosis in NSCLC cells is associated with alterations of the intracellular calcium signaling and disruption of mitochondria membrane integrity. Oncogene.

[74] Kang X., Kong F., Huang K., Li L., Li Z., Wang X., Zhang W., Wu X. (2019). LncRNA MIR210HG promotes proliferation and invasion of non-small cell lung cancer by upregulating methylation of CACNA2D2 promoter via binding to DNMT1. Onco Targets Ther..

[75] Jin R., Peng L., Shou J., Wang J., Jin Y., Liang F., Zhao J., Wu M., Li Q., Zhang B. (2021). EGFR-Mutated Squamous Cell Lung Cancer and Its Association With Outcomes. Front. Oncol..

[76] Wang C., Yin R., Dai J., Gu Y., Cui S., Ma H., Zhang Z., Huang J., Qin N., Jiang T. (2018). Whole-genome sequencing reveals genomic signatures associated with the inflammatory microenvironments in Chinese NSCLC patients. Nat. Commun..

[77] Cancer Genome Atlas Research N. (2012). Comprehensive genomic characterization of squamous cell lung cancers. Nature.

[78] Becirovic E. (2022). Maybe you can turn me on: CRISPRa-based strategies for therapeutic applications. Cell. Mol. Life Sci..

[79] Li T., Yang Y., Qi H., Cui W., Zhang L., Fu X., He X., Liu M., Li P.F., Yu T. (2023). CRISPR/Cas9 therapeutics: Progress and prospects. Signal Transduct. Target. Ther..

[80] Miserocchi G., Bocchini M., Cortesi M., Arienti C., De Vita A., Liverani C., Mercatali L., Bravaccini S., Ulivi P., Zanoni M. (2023). Combining preclinical tools and models to unravel tumor complexity: Jump into the next dimension. Front. Immunol..

[81] Durinikova E., Buzo K., Arena S. (2021). Preclinical models as patients’ avatars for precision medicine in colorectal cancer: Past and future challenges. J. Exp. Clin. Cancer Res..

[82] Zhou F., Zhou C.C. (2015). Targeted therapies for patients with advanced NSCLC harboring wild-type EGFR: What’s new and what’s enough. Chin. J. Cancer.

[83] Helissey C., Champiat S., Soria J.C. (2015). Immune checkpoint inhibitors in advanced nonsmall cell lung cancer. Curr. Opin. Oncol..

