# Supplementary material for: The X-Linked Tumor Suppressor TSPX Regulates Genes Involved in the EGFR Signaling Pathway and Cell Viability to Suppress Lung Adenocarcinoma
Source: Genes (Basel). 2025 Jan 11;16(1):75. doi: 10.3390/genes16010075 (PMC11764513; doi:10.3390/genes16010075)
Supplement: Supplementary file 1 [file genes-16-00075-s001.zip › genes-3386179-figures.pdf]

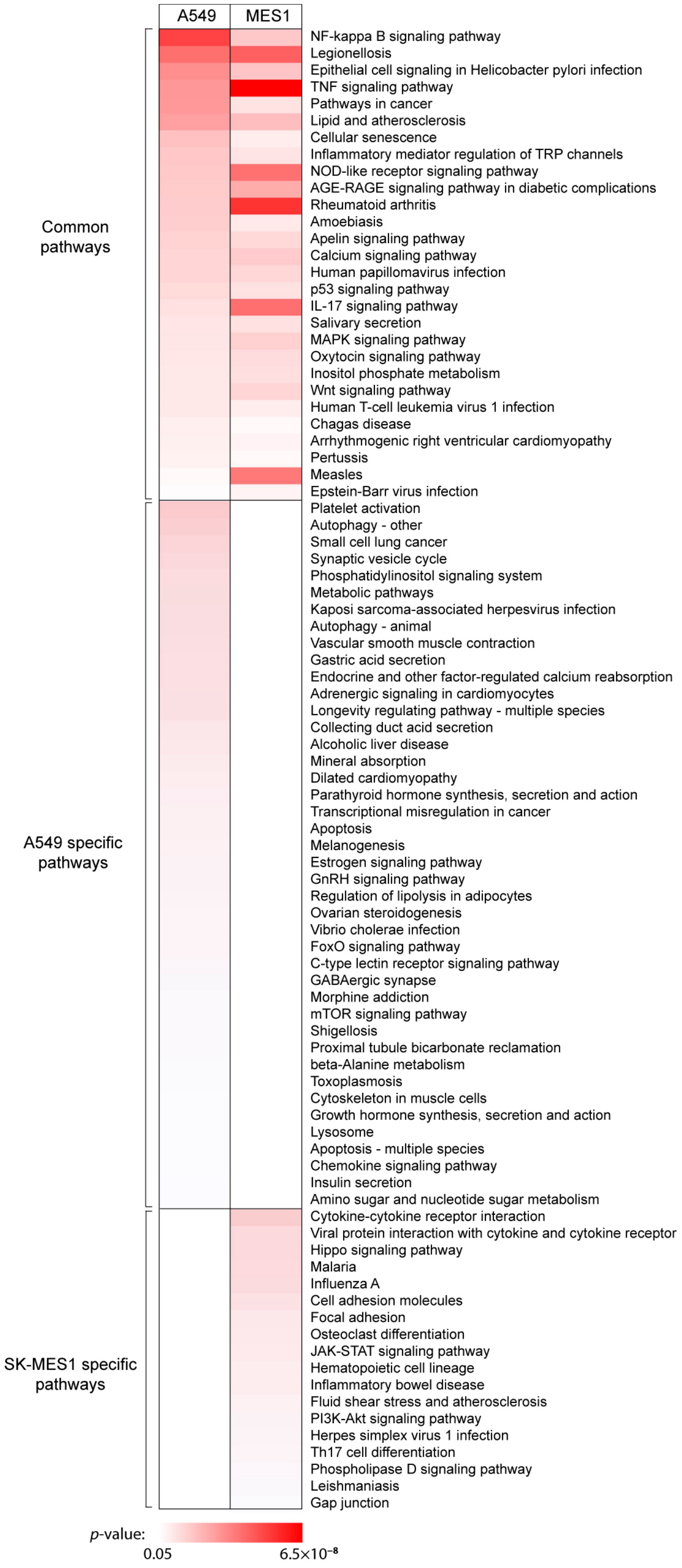

Figure S1. Enlarged image of the DAVID pathway enrichment analysis results for differentially expressed genes in TSPX-overexpressing A549 and SK-MES-1 cells.

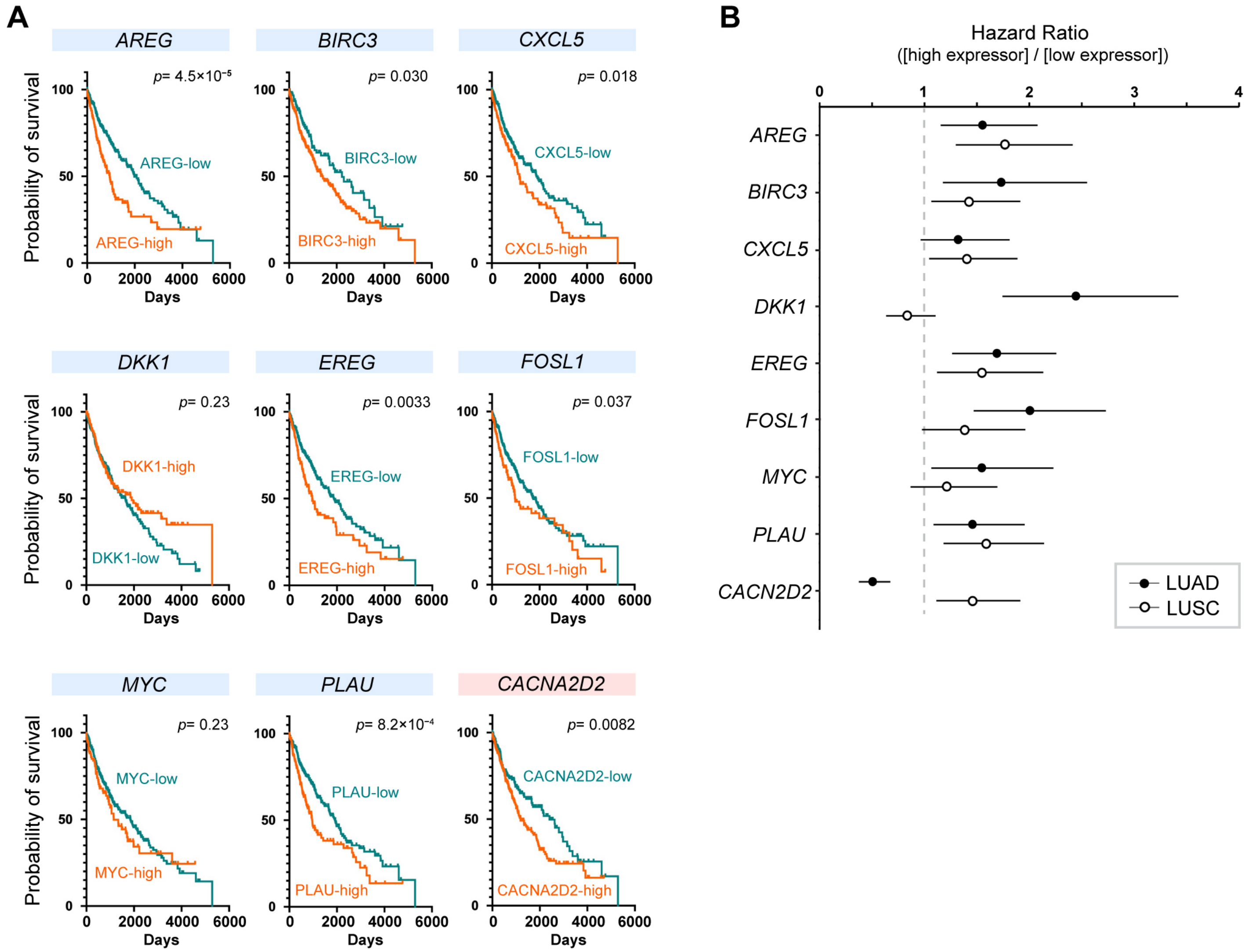

Figure S2. Correlations between the expression levels of TSPX downstream genes and patient survival in the TCGA lung squamous cell carcinoma dataset (TCGA-LUSC). (**A**) Correlation between the expression levels of the indicated genes and patient survival. Survival curves for high expressors (orange) or low expressors (green) are shown. Log-rank test *p*-values were obtained from TCGA datasets via the Human Protein Atlas (HPA) data portal. (**B**) The log-rank hazard ratios from survival analyses comparing the TSPX-high and TSPX-low groups. Filled circles indicate lung adenocarcinoma (LUAD) and open circles indicate lung squamous cell carcinoma (LUSC).
